# Plasticity of DNA methylation in mouse T cell activation and differentiation

**DOI:** 10.1186/1471-2199-13-16

**Published:** 2012-05-29

**Authors:** Yan Li, Guobing Chen, Lina Ma, Stephen J Ohms, Chao Sun, M Frances Shannon, Jun Y Fan

**Affiliations:** 1 College of Animal Science & Technology, Northwest A&F University, Yangling Shaanxi 712100, P. R. China; 2 Department of Genome Biology, John Curtin School of Medical Research, The Australian National University, Canberra ACT 2601, Australia; 3 ACRF Biomolecular Resource Facility, John Curtin School of Medical Research, The Australian National University, Canberra ACT 2601, Australia; 4 The University of Canberra, Canberra ACT 2602, Australia

**Keywords:** DNA demethylation, T cell activation, T cell differentiation, *Il2, Csf2*

## Abstract

**Background:**

Circulating CD4^+ ^T helper cells are activated through interactions with antigen presenting cells and undergo differentiation into specific T helper cell subsets depending on the type of antigen encountered. In addition, the relative composition of the circulating CD4^+ ^T cell population changes as animals mature with an increased percentage of the population being memory/effector type cells.

**Results:**

Here, we report on the highly plastic nature of DNA methylation at the genome-wide level as T cells undergo activation, differentiation and aging. Of particular note were the findings that DNA demethylation occurred rapidly following T cell activation and that all differentiated T cell populations displayed lower levels of global methylation than the non-differentiated population. In addition, T cells from older mice had a reduced level of DNA methylation, most likely explained by the increase in the memory/effector cell fraction. Although significant genome-wide changes were observed, changes in DNA methylation at individual genes were restricted to specific cell types. Changes in the expression of enzymes involved in DNA methylation and demethylation reflect in most cases the changes observed in the genome-wide DNA methylation status.

**Conclusion:**

We have demonstrated that DNA methylation is dynamic and flexible in CD4+ T cells and changes rapidly both in a genome-wide and in a targeted manner during T cell activation, differentiation. These changes are accompanied by parallel changes in the enzymatic complexes that have been implicated in DNA methylation and demethylation implying that the balance between these opposing activities may play a role in the maintaining the methylation profile of a given cell type but also allow flexibility in a cell population that needs to respond rapidly to environmental signals.

## Background

DNA methylation is one of the best characterized epigenetic modifications and changes in DNA methylation are associated with many biological processes. In higher organisms, a cell's phenotype, arising from alternative gene expression profiles, can be controlled at least in part by DNA methylation [1]. To date, the most accepted hypothesis is that gene silencing is correlated with DNA methylation at the promoter regions of the genes, while hypomethylation at such regions is linked to gene activation. Genomic DNA methylation is generally stable in somatic cells but is known to undergo extensive reprogramming at various stages of mammalian development [2-5]. There are many studies demonstrating distinct methylation levels and profiles in different cell types [6-10]. For example, a study focused on identifying the differentially methylated regions (DMRs) between conventional CD4^+ ^T cell (Tconv) and regulatory T cell (Treg) identified more than 100 DMRs that are present mainly at cell type-specific genes, such as *FOXP3 *and *IL2RA *[9]. Recent studies have provided strong evidence that DNA methylation is much more dynamic and flexible than previously believed and have identified mechanisms of active DNA demethylation in addition to the well-described DNA methylating mechanisms [11-14]. One recent study employing bisulphite conversion-based MethylC-seq, has identified promoter-distal regions as the major site of dynamic changes in DNA methylation levels during ES cell differentiation [15]. Another study employed a customized genome-wide methylation profiling method dubbed CHARM (comprehensive high-throughput arrays for relative methylation), and discovered waves of change in DNA methylation at 4.6 million potentially methylated sites during normal blood cell differentiation [16]. These and other studies provide evidence that DNA methylation of the mammalian genome is not only cell-type specific but also highly flexible and dynamic.

Circulating mature CD4^+ ^T cells are a highly flexible, mixed and dynamic population. The CD4^+ ^T cell population is a mixture of naive (CD44^-^CD62L^+^), memory/effector (CD44^+^CD62L^+/-^) and regulatory cells (CD4^+^CD25^+^). The relative proportions of these circulating cell types change naturally with age and upon encountering antigens. In responding to activation through interaction with antigen presenting cells, CD4^+ ^T cell undergo differentiation into numerous effector T helper cell types, such as Th1, Th2 and Th17, depending on the type of antigen encountered. As determined by the cell surface phenotypes and their functional characteristics, an increased proportion of T cells in elderly individuals is found to be highly differentiated as a consequence of repeated exposure to antigens and decreased production of naive T cells due to reduced thymopoiesis [17,18]. These events lead to a greater proportion of the CD4^+ ^T cells displaying an effector/memory phenotype. There have been numerous studies examining the role of DNA methylation in T cell activation and differentiation with a specific focus on individual genes. There is evidence that the level of DNA methylation changes at specific genes following T cell activation and differentiation. For example, reduced DNA methylation and stable DNase I hypersensitivity sites are observed at the *Ifng *and *Il4 *loci during Th1 and Th2 cell differentiation, respectively [19,20] and DNA methylation at the *FOXP3 *locus is greatly diminished in Treg cells compared to undifferentiated naive T cells [21-25]. A study investigating the role of CD44 in EAE (experimental autoimmune encephalitomyelitis) revealed that CD44 promotes Th1/Th17 cell differentiation with hypomethylation at the *Ifng *and *Il17a *promoters, whereas deficiency of CD44 inhibits Th1/Th17 cell differentiation and simultaneously enhances Th2/Treg cell differentiation with hypermethylation of *Ifng *and *Il17a *and hypomethylation of *Il4 *[26]. Thus, there is ample evidence supporting the idea that DNA demethylation or hypomethylation occurs at individual genes during the process of T cell differentiation.

It has also been well demonstrated that there are major changes in gene expression within a short time of the commencement of T cell activation and the role of associated changes in chromatin structure and histone modifications have been described [27]. A more surprising result was reported several years ago in relation to rapid changes in DNA methylation following T cell activation [28]. The promoter-enhancer region of the interleukin 2 gene was shown to undergo DNA demethylation within 20 minutes of stimulation in a transgenic T cell model [28]. This demethylation was shown to be DNA replication-independent and the authors proposed a possible active enzymatic mechanism although there were no clear candidate enzymes at that time [28]. Using reporter plasmids, DNA demethylation was confirmed to be necessary and sufficient to enhance transcription of *Il2 *[28]. Another study has shown that DNA demethylation at a specific CpG site at the promoter-enhancer of *IL2 *promotes the recruitment of *OCT*1 to its binding site and is regarded as a key epigenetic event in *IL2 *expression following CD4^+ ^T cell activation [29].

DNA methylation is also known to be an age-dependent process occurring naturally in all cells/tissues in mammals. Hypermethylation is found in aging liver and sperm cells in rats and in humans [30-32]. In contrast, age-dependent hypomethylation in interspersed repetitive sequences is also observed [33]. T cells produce less IL2 with age, and thus their function in elderly individuals is impaired [34,35]. On the other hand, decreased expression of the methyltransferases, *DNMT1 *and *DNMT3a*, is associated with increased expression of certain other genes in T cells during aging [36]. Although age-related DNA methylation in T cells is less well-studied, several studies have focused on the changes in DNA methylation in memory T cells. Production of cytokines, such as IL2, is increased in memory CD8^+ ^T cells compared with naive cells and is correlated with DNA demethylation occurring at the promoters of these cytokine genes [37,38]. These studies have all provided evidence that DNA methylation status, specifically at the promoters of inducible genes, is altered during T cell differentiation, activation and development.

DNA methylation and demethylation are two opposing epigenetic processes in genome regulation and the balance between them may help determine the level of genomic methylation in a given cell type. The DNA methyltransferases are part of the DNA methylation machinery required for maintaining genome stability through methylation of appropriate genomic regions [39]. These DNA methyltransferases, namely DNMT1, DNMT3a and DNMT3b, not only act as enzymes for maintaining genomic DNA methylation, but are also regarded as mediators of gene transcriptional silencing [40].

In addition to the DNA methyltransferases, the discovery of novel enzymes involved in active DNA demethylation has led to the idea that there may be a balance in the methylating and demethylating enzymatic activities that determines the level of genomic DNA methylation in any given cell. Although DNA demethylation was initially thought to occur only during DNA replication [41], active DNA demethylation has now been shown to occur independently of DNA replication and is involved in many developmental and physiological processes in animals and plants [11,12,14]. In plants, active DNA demethylation is important in controlling the activity of the RNA-directed DNA methylation pathway, and thus preventing the spread of methylation from repetitive sequences to their neighbouring genes [12]. In animals, several enzymatic complexes have recently been linked with active DNA demethylation [11,14]. Deaminases, including the AICDA and APOBEC family, are enzymes that catalyse the removal of -NH_2 _from DNA, and are responsible for hypermutation of their target substrates [42,43]. Glycosylases, such as Methyl-CpG-binding protein 4 (MBD4) and thymine DNA glycosylase (TDG), have also been reported to be involved in base excision repair and repair of T:G mismatches. MBD4 is one of the known thymine glycosylases and binds preferentially to 5mCpG · TpG mismatches, followed by efficient removal of thymine or uracil from a mismatched CpG site *in vitro *[44]. These enzymatic activities are now thought to act in a novel pathway of DNA demethylation [14]. A member of the Gadd45 family, Gadd45a, promotes the coupling process of an enzyme complex containing a cytosine deaminase, AICDA and a glycosylase, MBD4 [43]. An interesting study using *Gadd45b *knockout mice has recently revealed that GADD45b is required for activation-induced DNA demethylation of specific promoters and expression of corresponding genes critical for adult neurogenesis, including brain-derived neurotrophic factor and fibroblast growth factor [45].

DNA demethylation also occurs during embryonic stem (ES) cell differentiation via the Ten-eleven-translocation enzymes (TET1, TET2 and TET3) that catalyse the conversion of 5-methylcytosine (5mC) to 5-hydroxymethylcytosine (5hmC) [46,47]. In addition to 5hmC, the TET enzymes are also capable of generating other minor products, such as 5-formylcytosine (5fC) and 5-carboxylcytosine (5caC) [48]. 5caC can be specifically recognised and repaired by thymine DNA glycosylase (TDG) [49], one the enzymes involved in the GADD45/deaminase/glycosylase pathway mentioned above.

Given that the DNA methylation status of certain genes in T cells changes rapidly following cell activation and that differentiation leads to significant shifts in the gene expression profile, we have examined the changes that occur in DNA methylation at both the gene-specific and genome-wide level following activation and differentiation and have attempted to correlate this with changes in the enzymes known to be involved in DNA methylation and active demethylation.

## Results

### Global DNA demethylation occurs during T helper cell activation and differentiation into specific cell subsets

To examine the global changes in DNA methylation during T cell activation and differentiation, CD4^+ ^T cells isolated from young and adult mice were examined immediately following their activation and again following differentiation into T helper cell subsets. McrBC endonuclease was used to cleave the methylated genomic DNA at loci where pairs of (A/G)^Me^C were located between 40 and 3000 bp apart (^Me^C is methyl-cytosine on one or both strands of DNA).

**Figure 1:**
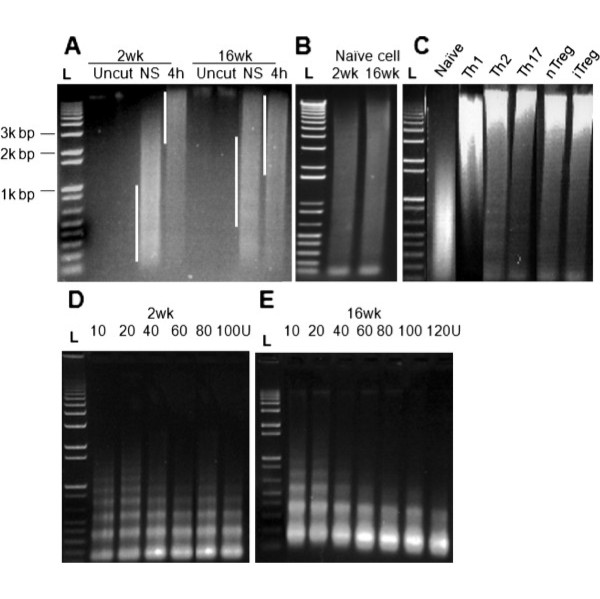
**Global DNA methylation and chromosomal accessibility in CD4^+ ^T cells**. *A. - C*. Genomic DNA isolated from total CD4^+ ^T cells taken from the spleens of 2 week or 16 week old C57BL/6 mice that were either unstimulated (NS) or stimulated with PMA/I for 4 hours (*A*), FACS-sorted naive T cells from both age groups (*B*), or FACS-sorted naive T cells and nTreg cells or differentiated Th1, Th2, Th17, and iTreg cells (*C*) were digested with McrBC for 6 hours at 37°C and the DNA fragments were resolved on a 1% agarose gel. Uncut genomic DNA is also shown. *D. & E*. Nuclei isolated from CD4^+ ^T cells of 2 weeks (*D*) and 16 weeks (*E*) old C57BL/6 mice were digested with various concentrations of MNase (10 U to 120 U) at 37°C for 5 min, prior to loading onto a 1% agarose gel for separation. The DNA was visualised by ethidium bromide staining in all experiments. The 1 kb plus ladder is indicated by the letter L. Every experiment was repeated at least 3 times

Genomic DNA isolated from total CD4^+ ^T cells of young (2 weeks old) and adult mice (16 weeks) either unstimulated or stimulated by PMA/I, was digested with McrBC at 37°C for 6 hours. In the unstimulated cells, clear differences in DNA methylation levels were observed between the two age groups (Figure 1A), as judged by the longer cleavage products after McrBC digestion in the older cells. In the 2 week-old group, the digested DNA fragments were about 200-1000 bp in length compared to 600-2000 bp at 16 weeks. Following PMA/I stimulation, DNA methylation levels decreased in both age groups compared to their unstimulated counterparts, but the change was more pronounced in the younger mice. In the 2 week-old group, the length of the digested DNA fragments increased significantly from 200-1000 bp in NS cells to over 3000 bp after 4 hours of stimulation; while in the 16 week group, the digested DNA fragments increased from 600-2000 bp in NS cells to over 2000 bp after 4 hours of stimulation. These results suggest that the DNA of T cells from older mice is less methylated than that of younger mice and that demethylation occurs early after stimulation by PMA/I in both groups of mice but is more pronounced in young mice. Subtle differences in the DNA methylation levels were also observed in the stimulated T cells in both age groups.

It has been shown that the developmental process leads to a change in the composition of the CD4^+ ^T cell population with an increase in the proportion of memory/effector T cells [50,51]. As seen in the FACS analysis (Additional file 1: Figure S1A, B), a shift from naive T cells to larger memory and effector T cell subset populations was observed during developmental progression. Naive T cells (CD44^-^CD62L^+^) were estimated at 90% (Additional file 1: Figure S1A) in the young mice but decreased to 64% in the adult mice (Additional file 1: Figure S1B), while effector/memory T cells (CD44^+^CD62L^+/-^) conversely increased from 4% to around 23% (Additional file 1: Figure S1A, B). We speculated that the difference in global DNA methylation levels between young and adult T cells detected in the McrBC assay might be related to the increase in memory and effector T cell populations in the older mice.

To confirm whether the change in DNA methylation levels was indeed related to the change in T cell subset populations, we performed similar McrBC experiments with FACS-sorted naive T cells isolated from both age groups. The cleavage products from McrBC endonuclease digestion showed a similar size distribution in both 2 week and 16 week naive T cells DNA (Figure 1B), indicating that genomic DNA isolated from naive T cells of both age groups had similar DNA methylation levels. Thus, the change in the proportion of memory/effector to naive T cells as mice age is likely contributing to the overall change in DNA methylation in the CD4^+ ^T cell population.

To determine whether differentiation of CD4^+ ^T cells *in vitro *leads to changes in the global DNA methylation, FACS-sorted naive T cells were stimulated under different polarising conditions using the appropriate cytokine combinations to generate specific T helper cell populations including Th1, Th2, Th17 and iTreg cells. The expression of genes specific for each of these T cell subsets was validated in each subset (Additional file 2) before the McrBC assay was used to determine global DNA methylation levels (Figure 1C). nTreg cells were also isolated from mouse spleens and the expression of *Foxp3 *used as a validation gene (Additional file 2). Interestingly, DNA demethylation occurred in all the differentiated T cell types, as judged by the fact that the digested products were significantly longer in the differentiated T cells after McrBC digestion compared with undifferentiated CD4^+ ^T cells. Thus, we conclude that global DNA demethylation is a common phenotype occurring during T cell differentiation.

Overall, it was observed that the degree of genome methylation declined significantly following T cell activation and differentiation. Naive T cells, therefore, appear to display a more hypermethylated phenotype while activated, differentiated or memory/effector T cells display a more hypomethylated phenotype.

Our previous studies showed that chromatin structure is altered after T cell activation and becomes more accessible in specific genomic regions linked to genes that respond to activation [27,52,53]. To determine whether chromatin accessibility changed with T cell development, CD4^+ ^T cells were isolated from both age groups of mice and digested with micrococcal nuclease (MNase). As expected, digestion with MNase produced nucleosomal ladders for both samples. Interestingly, an additional amount of micrococcal nuclease was required to digest the chromatin to mono- and di-nucleosomes in young mice (>100 units, Figure 1D), while a lesser amount of MNase was required for the adult mice (60 units, Figure 1F). This observation suggested that the global chromatin structure in the young mice was more compacted and less accessible to MNase. This compacted chromatin structure in the young mice correlates with the higher level of global DNA methylation (Figure 1A). Such compaction and hypermethylation in young T cells may be essential to maintaining inducible genes in an inactivated state in resting naive cells.

### DNA demethylation occurs in the promoters of the *Il2 *and *Csf2 *genes following CD4^+ ^T cell activation

**Figure 2:**
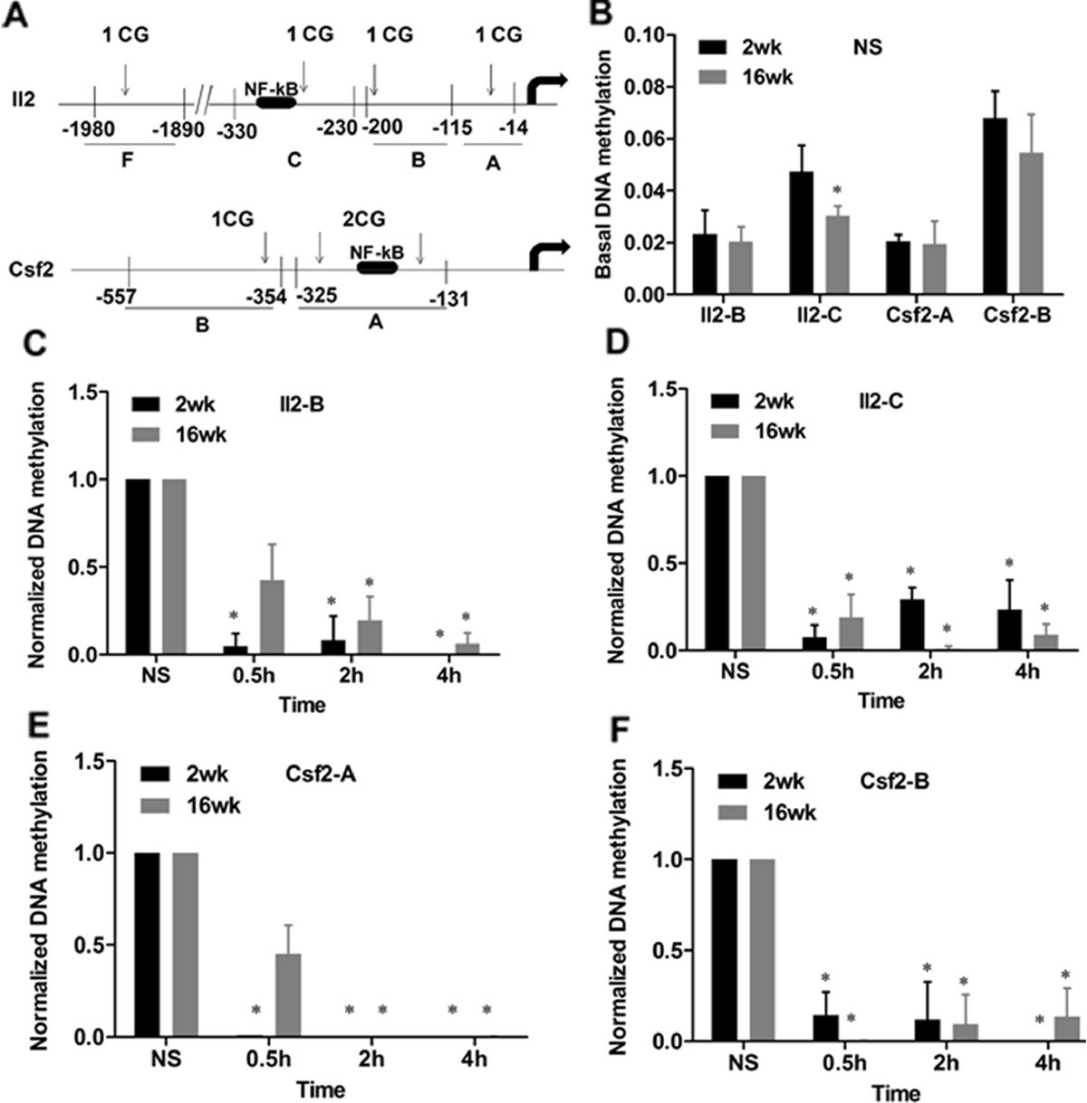
**DNA methylation changes at the promoters of *Il2 *and *Csf2 *during T cell activation and development**. *A*. Schematic representation of the promoter regions of the Il2 and *Csf2 *genes, showing the nucleotide distances upstream from the transcription start sites (dark arrows), the locations of the CpG dinucleotides (vertical arrows) and NF-kB binding elements for reference. PCR primers that covered one or multiple CpG dinucleotides were designed for each inducible gene for the MeDIP assay and are shown as thick horizontal lines and denoted by capital letters. *B. - F*. The MeDIP assay, using a 5-methylcytosine-specific monoclonal antibody (Epiteck), and real-time PCR, was used to determine the DNA methylation levels of individual CpG dinucleotides located at the promoter regions of *Il2 *and *Csf2 *in CD4^+ ^T cells. *B*. Basal DNA methylation levels at the promoter regions of *Il2 *and *Csf2 *in unstimulated CD4^+ ^T cells (NS) isolated from both age groups of mice were examined; the data are shown as means and SEM. *C. - F*. The DNA methylation levels of the *Il2 *and *Csf2 *genes in CD4^+ ^T cells upon PMA/I stimulation for 0, 0.5, 2 and 4 hours. The data were normalized to *Gapdh*, and were then normalized to the unstimulated samples, and are shown as the mean and SEM of three independent experiments. Every experiment was repeated at least 3 times

It has been shown previously, in a transgenic T cell model, that the *Il2 *gene promoter undergoes selective demethylation following cell activation [28]. We used direct immunoprecipitation of methylated DNA (MeDIP) [54,55] to examine whether DNA methylation at the promoters of the two inducible genes, *Il2 *and *Csf2*, changed in parallel with global changes in the total CD4^+ ^T cell populations isolated from both young and adult mice, either unstimulated or stimulated by PMA/I. The positions of the CG dinucleotides at the promoters of *Il2 *and *Csf2*, and the primers used for the DNA methylation study are illustrated in Figure 2A. Basal mRNA expression of *Il2 *and *Csf2 *was significantly higher in CD4^+ ^T cells from adult mice than younger mice (Additional file 3: Figure S3A). Therefore, we compared DNA methylation levels at the promoters of *Il2 *and *Csf2*, in the unstimulated CD4^+ ^T cells isolated from the two age groups. Similar DNA methylation levels were observed in these promoter regions in both young and adult mice with a small but significant decrease observed in the adult mice for one primer set only, i.e. primer set C of *Il2 *located across the NF-κB binding site (Figure 2B).

Following stimulation by PMA/I, a shift from naive T cells to larger memory and effector T cell subset populations was observed as seen in the FACS analysis (Additional file 1: Figure S1C, D). *Il2 *and *Csf2 *mRNA expression increased significantly in both age groups, although the fold change relative to the unstimulated T cells was more pronounced in the younger mice (40000-fold in young mice compared to 800-fold in adult mice for *Il2*, and 103-fold in young mice compared to 62-fold in adult mice for *Csf2 *at 4 hours post stimulation) (Additional file 3: Figure S3B, C). We next examined the relative changes in DNA methylation at the CG dinucleotides located at the promoters of the *Il2 *and *Csf2 *genes in response to cell stimulation (Figure 2C-F). During PMA/I stimulation, DNA methylation levels consistently decreased across the promoter regions of both inducible genes in the two age groups. However, the decrease in DNA methylation was not as rapid in the adult mice, at least for some of the primer sets, compared to the younger mice (Figure 2C-F). The rapid and dynamic decreases in DNA methylation in the regions examined are consistent with the global DNA demethylation observed in response to PMA/I stimulation in both age groups observed earlier (Figure 1A), and also correlate with the significant increases in mRNA expression of *Il2 *and *Csf2 *in the activated cells. These results support the previous study in a transgenic T cell model in which rapid DNA demethylation was observed at the *Il2 *promoter [28].

Thus, although rapid DNA demethylation in the *Il2 *and *Csf2 *promoter regions correlates with the observed increase in gene expression when T cells are activated, the difference in gene expression between young and adult T cells is not accompanied by a corresponding change in promoter DNA methylation.

### Chromatin accessibility at the *Il2 *and *Csf2 *promoters changes with age

**Figure 3:**
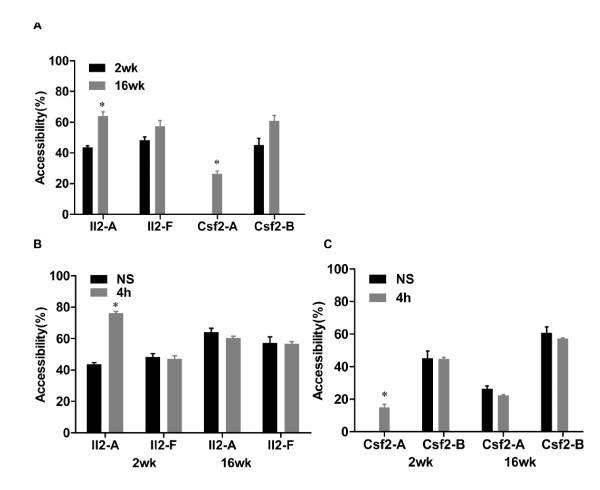
**Chromatin accessibility at the promoters of the *Il2 *and *Csf2 *gene promoters**. *A *- *C*. Nuclei isolated from unstimulated and stimulated CD4^+ ^T cells were digested with MNase (60 U) at 37°C for 5 min, and chromosomal accessibility at the promoter regions of *Il2 *and *Csf2 *in unstimulated CD4^+ ^T cells (NS) isolated from both age groups of mice (*A*), or at *Il2 *(*B*) and *Csf2 *(*C*) following T cell activation by PMA/I was determined by real-time PCR. Primer set A at the promoters of both cytokines, and *Il2 *set F located 2-kb upstream of *Il2 *TSS or *Csf2 *set B were located as shown in Figure 2A. DNA accessibility is calculated based on a method described previously [56]. Every experiment was repeated at least 3 times

We have previously shown that chromatin accessibility across the promoter regions of the *Il2 *and *Csf2 *genes changes significantly following T cell activation and correlates with an increase in gene expression and promoter function [53,56,57]. This study found that the major changes in MNase accessibility following stimulation of cells are limited to a region −60 to −200 bp upstream from the transcription start site[58], while a region closer to the transcription start site (−14 to −115) remains partially accessible after withdrawal of the simulus[56]. Thus we chose the promoter regions of *Il2*, set A/F and *Csf2*, set A/B to test local nucleosome accessibility using the CHART-PCR assay. Regions within the *Il2 *and *Csf2 *promoters (sets A) displayed greater accessibility in adult mice while regions outside the promoters (*Il2 *set F and *Csf2 *set B) displayed somewhat smaller changes in accessibility (Figure 3A). These results correlated with the higher basal expression of both genes in the adult T cells (Additional file 3: Figure S1A) but were not accompanied by a change in DNA methylation as demonstrated above (Figure 1B).

Similar to our previous studies [53,56], CD4^+ ^T cell activation by PMA/I resulted in higher MNase accessibility, which was particularly pronounced at primer sets A of both the *Il2 *and *Csf2 *promoters in the young mice (Figure 3B, C). The regions assayed by primer set F located 2 kb upstream of the TSS of the *Il2 *gene and primer set B located outside the core *Csf2 *promoter showed little change in MNase accessibility following activation in both age groups as expected from our previous studies. Most importantly, the lack of change in accessibility at primer sets A of the *Il2 *and *Csf2 *genes following activation of adult mice T cells may reflect a more open chromatin structure (Figure 3B, C) and the smaller fold change in gene expression may be due to the relatively higher basal levels of gene expression (Additional file 3: Figure S3B, C).

Promoter chromatin accessibility, therefore, correlates with gene expression levels whether during T cell development or following activation.

### DNA demethylation at individual gene promoters during T cell differentiation

**Figure 4:**
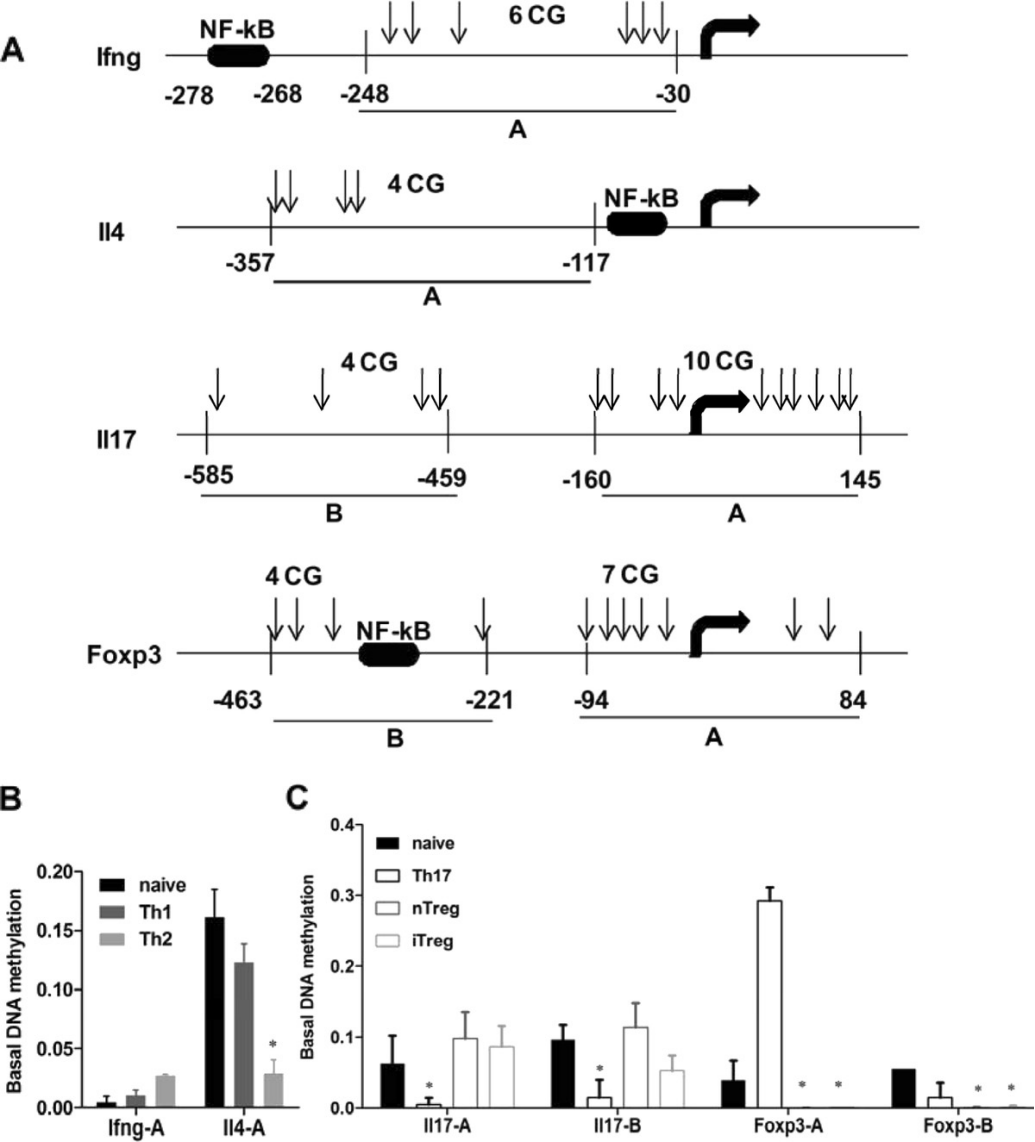
**DNA methylation changes at the promoter of signature genes in T cell differentiation**. *A*. Schematic representation of the promoter regions of *Ifng*, *Il4*, *Il17 *and *Foxp3 *genes, showing the nucleotide distances upstream from the transcription start sites (dark arrows), the locations of the CpG dinucleotides (vertical arrows) and NF-kB binding elements for reference. PCR primers that covered one or multiple CpG dinucleotides were designed for each inducible gene for the MeDIP assay and are shown as horizontal lines underneath the promoters and denoted by capital letters. *B*. Using a MeDIP approach, DNA methylation levels at the promoter regions of *Ifng *and *Il4 *(*B*), *Il17 *and *Foxp3 *(*C*) were examined by real-time PCR using the primer sets shown in *A*. The data were normalized to *Gapdh*, and were then normalized to the unstimulated samples, and are shown as the mean and SEM of three independent experiments

The experiments described above showed that global DNA demethylation occurred during differentiation of T cells in a number of different T helper cell subtypes. Thus we carried out MeDIP assays, using primers targeted to the promoter regions of specific genes in the undifferentiated naive and the differentiated Th1, Th2, T17, nTreg and iTreg cell subsets to determine whether DNA demethylation occurred at individual signature gene promoters during differentiation (Figure 4A). The *Ifng *promoter appeared to be hypomethylated in naive T cells and Th1 cells but methylation increased slightly in Th2 cells (Figure 4B). On the other hand, the *Il4 *promoter appeared to be heavily methylated in naive cells and Th1 cells and methylation levels significantly decreased following differentiation into Th2 cells (Figure 4B). These results agree with previous findings for these two genes [59-62]. DNA methylation levels in two regions of the *Il17 *promoter, a gene highly expressed in Th17 cells, were decreased in Th17 differentiated cells but not in iTreg or nTreg cells (Figure 4C). Conversely, DNA methylation in two regions of the *Foxp3 *promoter, a transcription factor that is a marker of Treg cells, was reduced in nTreg and iTreg cells but not in Th17 cells (Figure 4C). It should be noted that the level of DNA methylation in one region of the *Foxp3 *promoter was significantly increased in Th17 cells (Figure 4C) indicating that this gene may be targeted for repression.

These results indicate that although there is a significant change in overall or genomic DNA methylation as cells differentiate into T helper cell subtypes, there is also a level of specificity for demethylation at specific gene promoters.

### Changes in DNA methylating and demethylating enzymes in T cells

DNA methylation and demethylation are two opposing epigenetic processes involved in genome regulation and are modulated by various enzymes as described above. Since we observed changes in both global and gene-specific DNA methylation during T cell activation and differentiation, we were interested in determining whether the enzymatic mechanisms involved in these processes were also altered. Thus we investigated the mRNA expression of genes involved in the DNA methylation and demethylation pathways, including three DNMTs, the deaminase/glycosylase/GADD45 complex and the TET enzymes, following T cell activation and differentiation.

**Figure 5:**
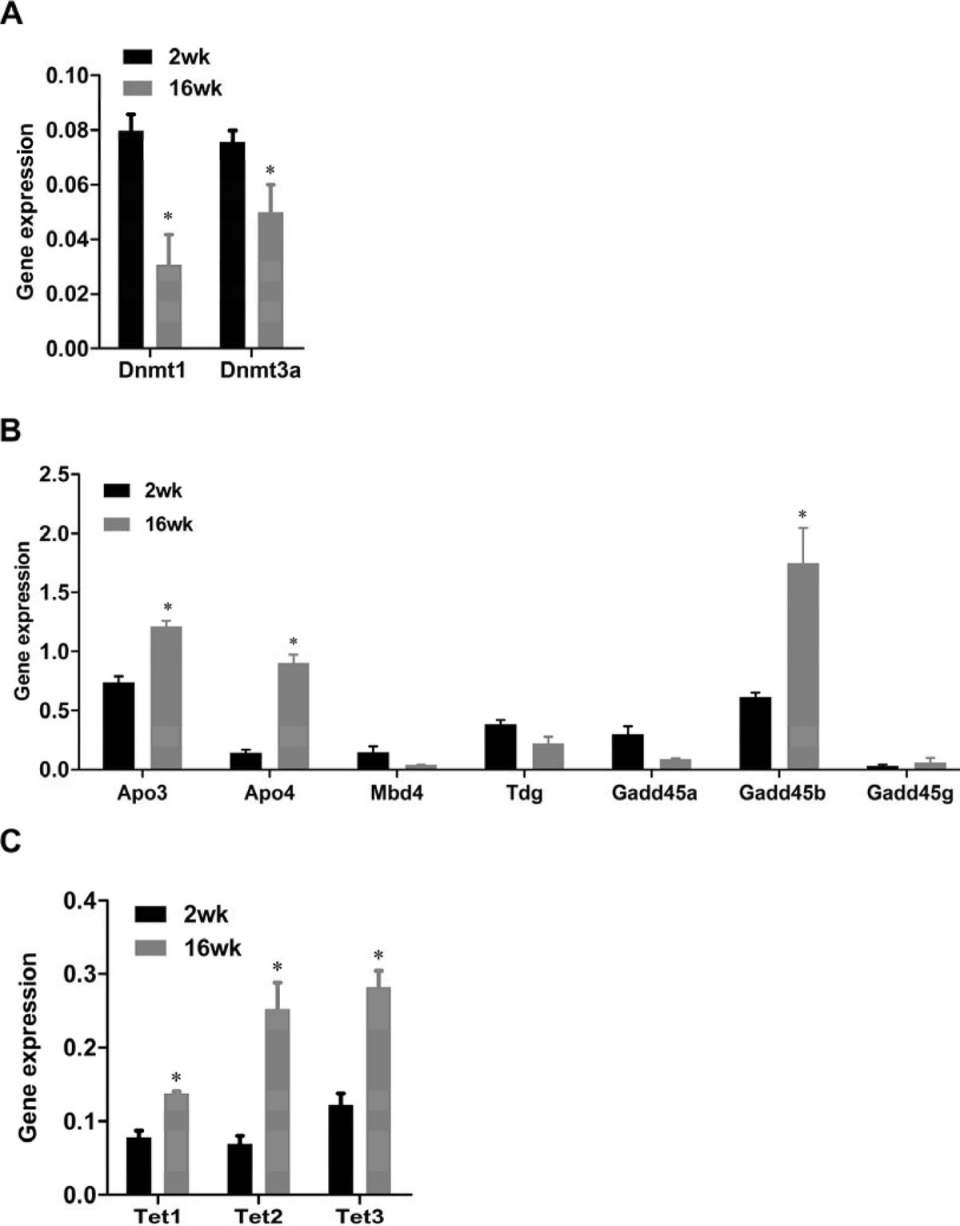
**mRNA expression of enzymes and co-factors involved in DNA demethylation in unstimulated CD4^+ ^T cells in two age groups of mice**. Total RNA was prepared from unstimulated CD4^+ ^T cells in both age groups of mice. After reverse transcription, quantitative PCR analysis was performed on cDNA with primers designed to detect these enzymes and co-factors. mRNA expression of (A) DNA methyltransferases (*Dnmt1*, *Dnmt3a*), (*B*) deaminases (*Apobec3 *and *Apobec4*), glycosylases (*Mbd4 *and *Tdg*), *Gadd45 *family (*Gadd45a*, *Gadd45b *and *Gadd45g*) and (*c*) ten-eleven translocation family (*Tet1*, *Tet2 *and *Tet3*) was determined by real-time PCR. The gene expression data were normalized to the *Ubc *housekeeping gene, and are shown as the mean and SEM of three independent experiments

The mRNA expression of both *Dnmt1 *and *Dnmt3a *was significantly reduced in the adult mice compared to young mice (p < 0.05) (Figure 5A) while *Dnmt3b *was barely detectable in cells of either age (data not shown). Elevated expression of both *Apobec3 *and *Apobec4 *was observed in adult mice compared to young mice as was the expression of *Gadd45b *(Figure 5B). In addition, a consistent increase in the expression of all three TET enzymes was observed in the older mice (Figure 5C). These data show that CD4^+ ^T cells from adult mice have reduced levels of enzymes that methylate DNA and higher levels of some members from two families of enzymes involved in active demethylation of DNA in other systems.

**Figure 6:**
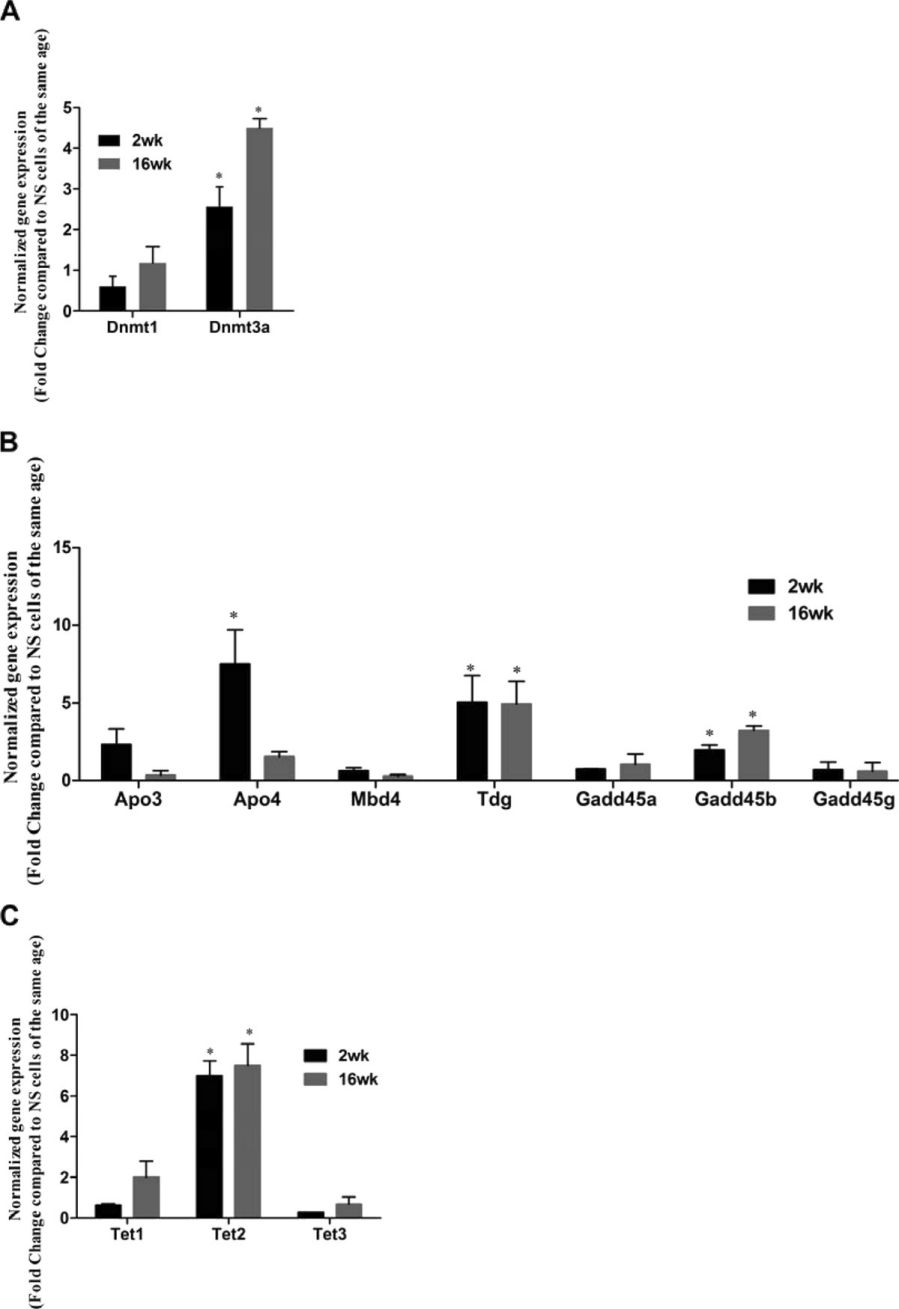
**mRNA expression of enzymes and co-factors involved in DNA demethylation in stimulated CD4^+ ^T cells**. *A - C*. mRNA expression of these enzymes and co-factors in CD4^+ ^T cells after 4 hours PMA/I stimulation in both age groups of mice. Expression is presented as fold change relative to unstimulated CD4^+ ^T cells (NS) *of the same age *in addition to initial *Ubc *normalization as mentioned earlier. The data are shown as the mean and SEM of three independent experiments

We next investigated the mRNA expression of the enzymes involved in DNA methylation and demethylation following activation with PMA/I stimulation in both age groups of mice. Expression of *Dnmt1 *was unchanged following stimulation, but we observed a greater than 2-fold increase of *Dnmt3a *expression in both age groups (Figure 6A). Also, the expression of *Dnmt3a *decreased after 8 h stimulation (data not shown). In addition, following stimulation, the mRNA expression of *Tdg *and *Gadd45b *increased in both age groups but *Apobec4 *was increased only in young mice (Figure 6B), This suggested that APOBEC4, TDG and GADD45b, might contribute to dynamic DNA demethylation processes during cell activation.

Interestingly, when we examined mRNA expression of the TET proteins in response to PMA/I activation, a greater than 7-fold increase was detected for *Tet2 *mRNA in both age groups (Figure 6C) suggesting that TET2 may be involved in DNA demethylation during T cell activation.

**Figure 7:**
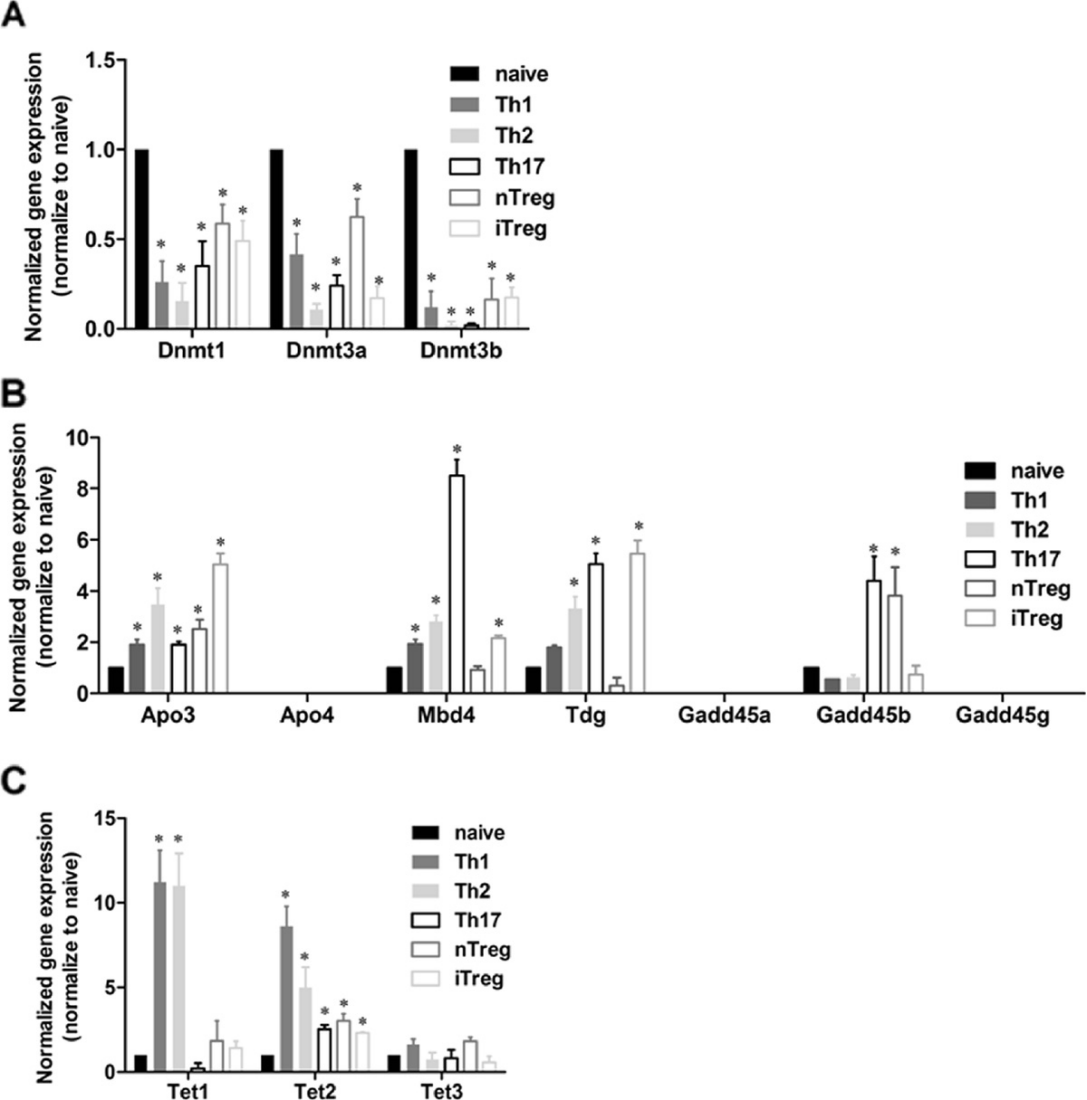
**mRNA expression of enzymes and co-factors involved in DNA demethylation in differentiated T cells**. *A. - C*. Comparison of mRNA expression of these enzymes and co-factors in non-differentiated naive T cells and differentiated T cells (Th1, Th2, Th17, nTreg and iTreg). The data were normalized to naive cells in addition to initial *Ubc *normalization as mentioned earlier, and are shown as the mean and SEM of three independent experiments

Lastly, we examined the mRNA expression of these enzymes in undifferentiated naive T cells and differentiated Th1, Th2, Th17, nTreg and iTreg cells (Figure 7A-C). The expression of all three DNA methyltransferases was reduced in each of the differentiated T cell subsets compared to the undifferentiated naive T cells (Figure 7A). *Apobec3*, *Mbd4 *and *Tdg *all displayed a greater than 2-fold increase in expression in all differentiated T cells, with the exception of nTreg cells (Figure 7B). Increased expression of *Gadd45b *(almost 6-fold) was detected only in the differentiated Th17 and nTreg cells (Figure 7B). Although the pattern of expression of these enzymes was variable among the differentiated T cell subsets, there was evidence that the demethylating enzyme complexes were generally more highly expressed in differentiated T cells.

When we examined expression of the TET genes, *Tet2 *was the only gene that showed a greater than a 2-fold increase in mRNA expression in all the differentiated T cells (Figure 7C). In addition, there was a greater than 10-fold increase in *Tet1 *expression in both Th1 and Th2 cells (Figure 7C), suggesting that both TET1 and TET2 may be involved in DNA demethylation during T cell differentiation processes.

Overall, our data revealed that enzymes involved in the two opposing epigenetic processes were dramatically altered during T cell activation and differentiation. GADD45 proteins along with deaminases, glycosylases and the TET proteins were significantly up-regulated in response to T cell activation and differentiation, while changes (generally decreases) were also observed for the DNA methyltransferases.

### 5-hydroxymethylcytidine is detected at gene promoters following T cell activation and differentiation

**Figure 8:**
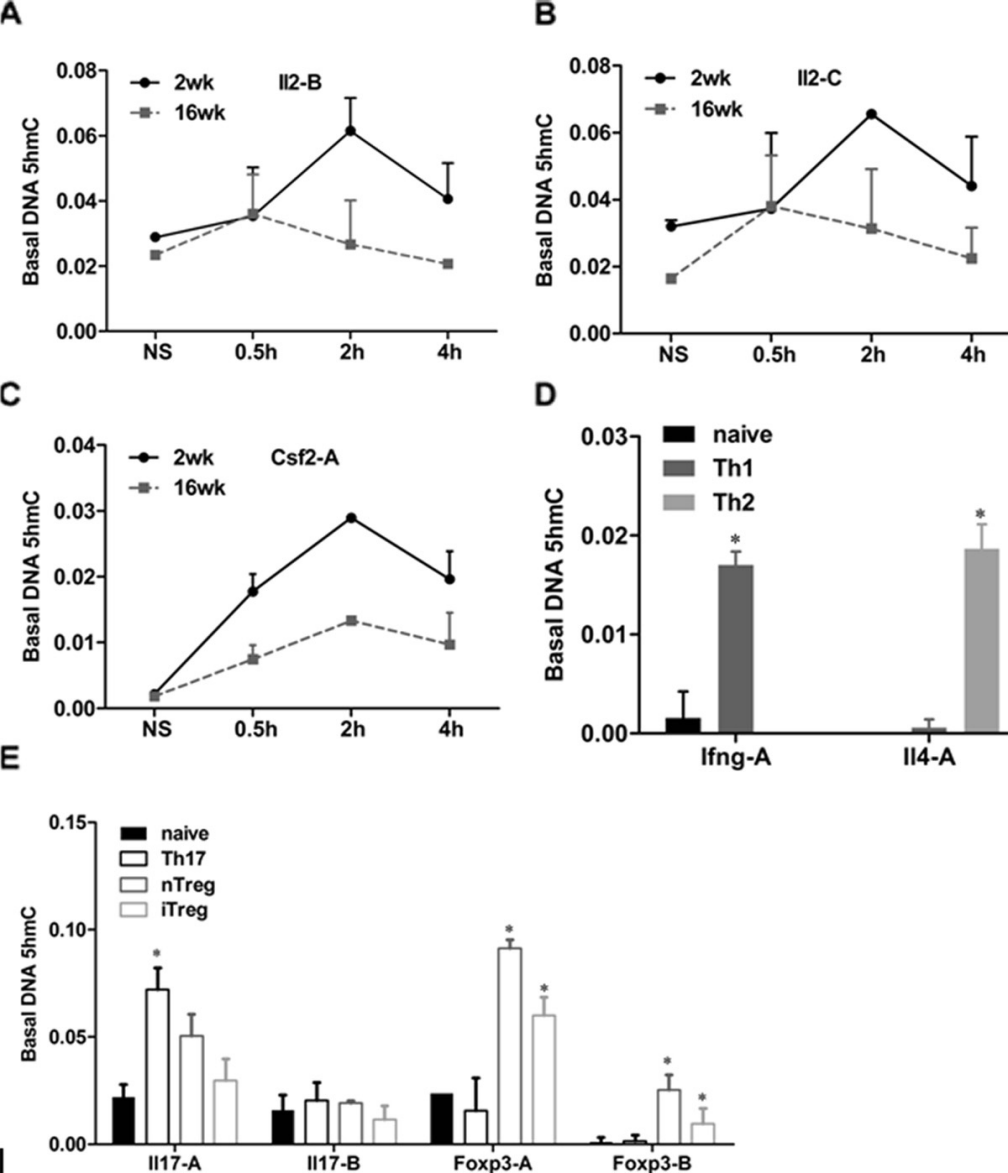
**Generation of 5-hydroxymethylcytosine at specific gene promoters in CD4^+ ^T cells**. *A. - E*. The MeDIP approach, using a 5-hydroxymethylcytosine (5hmC) antibody (Active Motif) that specifically recognizes 5-hydroxymethylcytosine, was applied to determine the DNA 5hmC levels at individual CpG dinucleotides located at the promoter regions of inducible genes in CD4^+ ^T cells from mice of both age groups during PMA/I stimulation for 0, 0.5, 2 and 4 hours (*A*. - *C*.) and T cell differentiation (*D. - E*.); primers *Il2 *set B (*A*) and *Il2 *set C (*B*) are located at the *Il2 *promoter; *C *. primer *Csf2 *set A is located at the *Csf2 *promoter; primer sets A of *Ifng *and *Il4 *are located at the *Ifng *and *Il4 *promoters, respectively; primers *Il17 *set A and set B, *Foxp3 *set A and set B are located at the *Il17 *and *Foxp3 *promoters, respectively. The data were normalized to NS, and are shown as the mean and SEM of three independent experiments

The TET family of enzymes play important roles in regulating DNA methylation in mouse embryonic stem (ES) cells, and can catalyse the conversion of 5-methylcytosine (5mC) to 5-hydroxymethylcytosine (5hmC) [46,47]. Therefore, the level of 5-hydroxymethylcytosine (5hmC) was determined at specific promoter regions by a methyl DNA immunoprecipitation assay using an antibody specifically recognising 5-hydroxymethylcytosine (5hmC). The level of 5hmC at both of the *Il2 *and *Csf2 *promoters following CD4^+ ^T cell activation was examined and consistent increases in the 5hmC product were observed in both age groups of mice after stimulation but were more pronounced in the younger mice (Figure 8A-C), correlating well with the dynamic DNA demethylation observed earlier following activation (Figure 2C-F). Distinct time courses were observed in young and adult cells as were observed for the demethylation time courses (Figure 2C-F). Increased 5hmC levels were also detected at the promoter regions of *Ifng *or *Il4 *in Th1 and Th2 cells, respectively, while low signals in these regions were detected in naive T cells (Figure 8D). Similarly, increased 5hmC levels were detected at the promoter regions of *Il17 *or *Foxp3 *in Th17 and Treg cells, respectively (Figure 8E), and are consistent with the decreased DNA methylation levels detected in these regions described earlier.

Thus, changes in 5hmC levels accompany changes in gene expression during T cell activation and differentiation and, together with the data showing a corresponding increase in expression of the *Tet *genes, suggest that the generation of 5hmC may be part of the mechanism of demethylation.

## Discussion

The results presented in this paper show that DNA methylation is dynamic in mouse T cells with distinct DNA methylation levels observed in different CD4^+ ^T cell populations. Following T cell activation, demethylation of DNA occurs rapidly with a major global shift observed within hours of stimulation. In addition, all the differentiated T cell types examined, including regulatory T cells, appear to have a more hypomethylated phenotype than their non-differentiated counterparts. CD4^+ ^T cells from older mice also display a hypomethylated phenotype but this appears to be related to the altered cellular composition of the older T cell population with a greater percentage of the cells being effector/memory type cells. These results are in agreement with many recent observations showing that DNA methylation is more dynamic than previously considered [10-12,14,63,64] and that changes in DNA methylation are not always linked to DNA replication [28]. DNA demethylation can occur in response to extracellular signals, during embryonic development and during tissue differentiation [5,14]. These changes in DNA methylation have been documented both at the global genome-wide level and at individual genes. For example, in experiments fusing human fibroblasts and mouse muscle cells to form non-dividing heterokaryons, active DNA demethylation and subsequent expression of the *Myod1 *gene was observed [65]. Active DNA demethylation, independent of cell division, has also been observed on the paternal genome but not the maternal genome following fertilisation [66,67]. Active DNA demethylation has also been demonstrated in post-mitotic neurons at the promoters of brain-derived neurotrophic factor (*Bdnf*) and fibroblast growth factor-1 (*Fgf1*) [68].

In this study, analysis of individual genes demonstrated that following T cell activation, there was very rapid demethylation at both the *Il2 *and *Csf2 *promoters, two genes whose expression is rapidly induced following activation. These results are in agreement with a previous study of the *Il2 *gene in a TCR transgenic mouse model [28] which clearly demonstrated that the demethylation observed at the *Il2 *promoter following T cell activation was rapid and independent of DNA replication. In addition, our results show that the rapid loss of 5-methylcytosine is accompanied by an increase in 5-hydroxymethycytosine at the promoter of both genes implying a role for the TET family [11,14] in an active mechanism of demethylation. This activation-dependent demethylation was observed in T cells from both young and adult mice although the degree and timing differed in the two groups.

Older T cells express more *Il2 *and *Csf2 *in the resting state than younger T cells, most likely due to the increased percentage of the population that has an effector/memory phenotype. Decreased expression of the methyltransferases, *DNMT1 *and *DNMT3a*, has been associated with increased gene expression in T cells during aging [36] and we also observed a significant decrease in these enzymes in older T cells. We also show here that the mRNA expression of several proteins implicated in DNA demethylation is increased in the older T cell population. Although there was a strong difference in global DNA methylation levels between younger and older T cells, in parallel with the changes in the expression of the methylating and demethylating enzymes, we detected only small differences between these cell populations at the *Il2 *or *Csf2 *promoters. We attribute this to the sensitivity of the assay since the naive cell population dominated the total CD4^+ ^T cell population. We also detected small differences in chromatin accessibility suggesting that these two genes were more active in the older population, an observation borne out by the increases observed in the mRNA levels. It is likely that these increases in mRNA are the result of increased expression in only a fraction of the total population being examined thus leading to difficulties in detecting changes in DNA methylation and chromatin accessibility in the total population.

Although we observed large changes in the global level of DNA methylation following T cell differentiation, we also observed a significant degree of specificity when individual genes were examined. For example, the *Il4 *promoter was demethylated in Th2 but not Th1 cells as has been previously demonstrated [19]. The differentiation of Th17 and Treg cells has been linked through a common TGFβ signalling pathway in which transcription factors such as RORγt and FOXP3 interact in a competitive antagonism and drive commitment towards the Th17 or Treg lineages respectively [69]. We observed a clear distinction in the demethylation patterns of the Th17-specific *Il17 *promoter and the Treg-specific *Foxp3 *promoter with the most significant demethylation observed in the cells that express these genes. These results imply that targeted demethylation occurs on specific genes expressed in, and linked to the function of these cell types. There is evidence that recruitment of DNA methyltransferases to specific genes may change upon T cell differentiation [60]. Under Th2 polarising conditions, DNA demethylation at *Il4 *and *Il13 *is initiated through reduced DNMT1 recruitment and an increase in active histone H3K4 methylation markers at this locus [60]. FOXP3 is a master transcription factor required for the development and function of Treg cells and the DNA methyltransferases, DNMT1 and DNMT3A, have been shown to be associated with the *Foxp3 *locus in CD4^+ ^T cells, resulting in repression of *Foxp3 *expression [70]. Recruitment of the opposing DNA demethylase complexes will now need to be examined.

Although enzymes involved in DNA methylation have long been known, a mechanism to explain active DNA demethylation has only recently been described [11,12,14]. A plethora of recent studies has demonstrated two, possibly interlinked, mechanisms of active DNA demethylation as described earlier [14]. Here, we examined the expression of the various enzymatic and non-enzymatic components of these DNA demethylating complexes, in addition to the well-described DNA methylation enzymes, to ask if there was a correlation between DNA methylation levels and the expression of these opposing enzymatic activities. We detected the expression of *Dnmt1*, *Dnmt3a *and *Dnmt3b *in CD4^+ ^T cells and found that expression was decreased in older T cells and in all differentiated T cell types in parallel with the globally hypomethylated phenotype. In parallel with the decrease in DNA methylating enzymes observed in differentiated T cells compared with their non-differentiated counterparts, we found increased mRNA expression of various members of the AICDA/APOBEC/glycosylase and TET families of demethylating enzymes. Of particular note is the strong increase observed for the *Tet1 *and *Tet2 *proteins in Th1 and Th2 cells and the accompanying increase in 5hmC at the promoters of the *Ifng *and *Il4 *genes in Th1 and Th2 cells respectively. The *Foxp3 *gene promoter also displayed a clear increase in 5hmC in both nTreg and iTreg cells although this was associated with a smaller increase in expression of the TET family members in these cell types. Each differentiated cell type appears have a distinct profile of expression of the AICDA/APOBEC/glycosylase family members as well as the GADD45 scaffold proteins suggesting that each cell type may use distinct demethylating complex(es) to achieve a more hypomethylated state.

Examination of the expression of the DNA demethylating complexes clearly showed that T cell activation led to an increase in the expression of several components of the AICDA/APOBEC complex, in particular, *Apobec3 *and *Apobec4*, as well as the DNA glycosylase *Tdg*. The expression of *Tet2*, a member of the TET family that can hydroxylate 5mC to 5hmC was also increased following T cell activation and as discussed above, there was an increase in 5hmC observed at the promoters of both the *Il2 *and *Csf2 *genes. Members of the GADD45 family have been implicated in the function of the AICDA/APOBEC family by acting as a scaffold protein to coordinate the deamination and glycosylase functions [45]. GADD45 proteins have long been implicated in immune function [71-75] and the expression of *Gadd45b *increased following cell activation as in the previous studies [74,75]. *Dnmt3a *expression increased immediately following T cell activation, an unexpected result given the observed decrease in DNA methylation following activation. However the expression of *Dnmt3a *subsequently decreased after stimulation for 8 h (data not shown). Nevertheless, two clear points emerge from these studies. Firstly, when T cells are activated, differentiate or age (and hence change in cellular composition) there is a shift in the balance of expression of the DNA methylating and demethylating enzymes accompanied by a shift in the global methylation status of the DNA. Secondly, these activities must be targeted to specific genomic regions or genes whose expression changes during these processes and further studies need to be done to determine the targeting mechanisms.

DNA demethylation has been linked to autoimmunity, in particular to systemic lupus erythematosus (SLE). CD4^+ ^T cells from SLE patients have been shown to have significantly lower DNA 5mC content than T cells from controls [76,77] and mouse CD4^+ ^T cells treated with a methylation inhibitor and transferred to syngeneic mice induce symptoms of SLE [78]. The expression of specific genes, such as perforin, is increased in CD4^+ ^lupus cells and this is accompanied by hypomethylation at their promoters [79]. However, there has not been a clear demonstration of a decrease in the expression of the DNA methylating enzymes despite several studies [36,79]. A recent study, however, showed that GADD45a may contribute to autoimmunity by promoting DNA demethylation in lupus T cells [71]. This work raises the possibility that the active DNA demethylating enzyme complexes such as AICDA/APOBEC and the TET family may be associated with autoimmunity and warrants further investigation.

## Conclusions

In summary, we have demonstrated that DNA methylation is dynamic and flexible in CD4^+ ^T cells and changes rapidly both in a genome-wide and in a targeted manner during T cell activation and differentiation. These changes are accompanied by parallel changes in the enzymatic complexes that have been implicated in DNA methylation and demethylation implying that the balance between these opposing activities may play a role in the maintaining the methylation profile of a given cell type and also allow flexibility in a cell population that needs to respond rapidly to environmental signals.

## Materials and methods

### Animals and ethics

Male C57BL/6 mice (Animal Resources Centre (ARC)) were housed in a room at constant temperature (22 ± 1°C) with a 12-hour alternating light dark cycle (6:00-18:00), and given free access to commercially-produced mouse feed and tap water. This study was approved by the Animal Experimental Ethics Committee of the Australian National University (Animal Ethics Project Numbers J.MB.45.09 and J.MB.51.10).

### Cell culture and stimulation

Spleens were isolated from C57BL/6 mice (2 and 16 weeks old). CD4^+ ^T cells were purified in MLC medium using MACS CD4^+ ^(LT34) beads according to the manufacturer's guidelines (Miltenyi Biotec, Bergisch Gladbach, Germany). Pure CD4^+ ^T cells were cultured in completed MLC with 10% fetal bovine serum (FBS), 10 mM HEPES, 4 mM glutamine, 1 mM sodium pyruvate, 1.76 μL β-mercaptoethanol and antibiotics.

The CD4^+ ^T cells were stimulated by addition of phorbol 12-myristate 13-acetate (PMA) (10 ng/mL) and calcium ionophore (I) (1 μM). Cells were incubated inside a standard CO_2 _incubator at 37°C with 5% CO_2_.

### Th1 and Th2 cells

Naive T cells were initially isolated from C57BL/6 mice spleens (ARC, 7 weeks old), followed by sorting with flow cytometry using CD4^+ ^and CD62L^+ ^as selective markers. The sorted naive T cells were cultured initially in MLC medium supplemented with 10% heat-inactivated FCS, 2 mM L-glutamine, 100 IU/mL penicillin, 100 μg/mL streptomycin, and 50 μM β-mercaptoethanol (all from Sigma-Aldrich). For generation of Th1 or Th2 cells, 0.25 × 10^6 ^cell/mL naive T cells were cultured on anti-CD3 (2 μg/mL) and anti-CD28 (2 μg/mL) coated 24-well plates in the presence of either 10 ng/mL IL12 (R&D Systems), 10 μg/mL anti-IL4 (1B11) blocking antibodies for the Th1 condition; or 10 ng/mL IL4 (R&D Systems), 10 μg/mL anti-IL12 (p40/p70), 10 μg/mL anti-IFNg blocking antibodies for the Th2 condition. Both Th1 and Th2 cells were cultured under their corresponding conditions for a maximum of 8 days inside a standard CO_2 _incubator at 37°C with 5% CO_2_, with medium changed every two days. IL4 and IFNg production in the induced CD4^+ ^T cells were determined by intracellular cytokine staining as described later. Th1 and Th2 differentiated T cells were further enriched by FACS with CD4^+^IFNg^hi ^and CD4^+^IL4^hi ^as selective markers, respectively (Additional file 4: Figure S4A, B).

### Th17 cell polarization

Naive T cells (CD4^+^CD62L^+^) sorted by flow cytometry from 7 week old C57BL/6 mice spleens were cultured in MLC medium supplemented (detailed in Th1 and Th2 above), under Th17 polarising conditions with 10 μg/mL CD3e, 5 μg/mL CD28, 10 ng/mL IL6 (all from BD), and 1 ng/mL TGFβ as stimuli. All these reagents were directly added to the cells, without prior coating in 50 mL flasks. After stimulation for 4 days inside a standard CO_2 _incubator at 37°C with 5% CO_2_, cells were harvested and washed at least three times with cold 2% BSA-PBS. Th17 cells were captured with IL17 capture kit (BD) according to the manufacturer's instructions and the methods published previously [80,81]. Briefly, cells were incubated with 2 aliquots of IL17A capture complex (BD) in 500 μL cold medium at a maximum density of 8 × 10^6^/mL on ice for 10 min. Warm medium (40 mL, 37°C) was added and the mixture was incubated and gently mixed intermittently for an additional 1.5 hours at 37°C. After cytokine capture, extra cold 2% BSA-PBS was added and placed on ice for 15 min. Cells were spun down and washed twice before staining with PE-conjugated anti-IL17A followed by cell sorting. Cell viability was determined by trypan blue exclusion and was always greater than 90% after isolation (Additional file 4: Figure S4C).

### Natural Treg (nTreg) cells

Natural Treg cells (CD4^+ ^CD25^+ ^) were sorted by flow cytometry from 7 week old C57BL/6 mice spleens (Additional file 4: Figure S4D).

### Induced Treg (iTreg) cells

For generation of iTreg cells, naive T cells (CD4^+^CD25^-^) sorted by flow cytometry from 7 week old male C57BL/6 mice spleens, were stimulated with anti-CD3 (6 μg/mL) and anti-CD28 (4 μg/mL) coated 6-well plates in the presence of either 10 ng/mL IL2 (R&D Systems) or 5 ng/mL human TGFβ1 (R&D Systems) for 4 days, and in MLC medium supplemented with 10% heat-inactivated FCS, 2 mM L-glutamine, 100 IU/mL penicillin, 100 μg/mL streptomycin, and 50 μM mercaptoethanol (all from Sigma-Aldrich). FOXP3 production in total CD4^+ ^T cells was determined by intracellular cytokine staining as described below. iTreg differentiated T cells were further enriched by FACS with CD4^+ ^and CD25^+ ^as selective markers (Additional file 4: Figure S4E).

### Surface staining/FACS

CD4^+ ^T cells (at least 50,000 cells) were incubated with antibodies (1:200 dilution) in 200 μL PBS containing 2% BSA for 15-30 min at 4°C. Cell pellets were resuspended in 200 μL PBS containing 0.5% BSA and kept on ice until FACS.

### Intracellular staining

GolgiStop (0.7 μL/mL) was added to the stimulated CD4^+ ^T cells. The cells were first stained with FITC-CD4 (1:400) in PBS containing 2% BSA buffer on ice for 30 min, and then fixed in 200 μL permeabilization buffer for 30 min on ice. Stained cells were then washed twice with 200 μL of the same buffer, before incubating with CD16/CD32 (1:400) and 200 μL permeabilization buffers for 30 min on ice. The cells were finally stained with PE-IFNg, PE-IL4 or PE-IL17A for 30 min on ice, washed once with 200 μL permeabilization buffer, and twice with PBS containing 2% BSA.

### cDNA Synthesis and Real time-PCR

Total RNA was prepared from stimulated and unstimulated T cells using TriReagent (Sigma-Aldrich) according to the manufacturer's instructions. RNA (1 μg) was treated with DNase I (1 U) in Tris buffer (pH 7.6) containing 5 mM MgCl2 at 37°C for 30 min, and reverse-transcribed using first-strand cDNA synthesis (Marligen, Ljamsville, USA) as detailed in the manufacturer's instructions. Real-time PCR reactions were performed with 50 ng of cDNA and Power SYBR^® ^Green PCR Master Mix (ABI) in a total volume of 20 μL on an ABI 7500 sequence detector (Applied Biosystems). Details of the primers used for gene expression are listed in Additional file 5. The Ct values for the genes of interest were normalized to the housekeeping gene, Ubiquitin-conjugating enzyme E2D (*Ubc*), whose expression was not altered in response to PMA/I stimulation. The following PCR conditions were used: stage 1, 50°C for 2 min for one cycle; stage 2, 95°C for 10 min for one cycle; and stage 3, 95°C for 15 sec and 60°C for 1 min for 40 cycles.

### McrBC assay

Genomic DNA from CD4 T cells was extracted using a QIAamp DNA Blood Mini Kit (Qiagen) based on the manufacturer's protocol. Genomic DNA (1 μg) was digested with McrBC (10 units) endonuclease (NEB) in the presence of 10 mM GTP and NEBuffer 2. The sample was incubated at 37°C for 6 hours prior to loading onto a 1% agarose gel. After staining with ethidium bromide, gel images were taken with a Gene Genius Bio Imaging system (Syngene).

### MeDIP

Genomic DNA from CD4 T cells was extracted using a QIAamp DNA Blood Mini Kit (Qiagen). For MeDIP, gDNA (2 μg) was sonicated in an ice bath for 20 min at 4°C until average DNA fragments were in the range of 500 to 1000 bp. gDNA samples were denatured at 90°C for 10 min before incubation with 2 μg 5-methylcytosine antibody (Epigentek, 33D3) in IP buffer (100 mM sodium phosphate, pH 7.0, 1.4 M NaCl and 0.5% Triton X-100) for at least 1 hour at 4°C. Dynabeads^® ^Sheep-anti Mouse IgG (Invitrogen, 20 μL, pre-washed with PBS-BSA 0.1%) was incubated with the gDNA/5-methylcytosine antibody mixture for an additional 1 hour at 4°C. The Dynabeads were then collected on a magnetic rack, washed with IP buffer three times and TE buffer once, before resuspension in proteinase K digestion buffer (50 mM Tris, pH 8.0, 10 mM EDTA and 0.5% SDS) and 100 mg proteinase K and incubated at 65°C for 1 hour. Enriched methylated DNA fragments were further purified from the final mixture using PCR purification kits (Invitrogen). Real-time PCR reactions were performed with the enriched DNA fragments and Power SYBR^® ^Green PCR Master Mix (ABI) in a total volume of 20 μL on an ABI 7500 sequence detector (Applied Biosystems). Details of the primers used for the gene expression are listed in Additional file 6.

### CHART assay

Cell pellets (5 × 10^7 ^cells) were resuspended gently in 10 mL buffer A (10 mM Tris, PH 7.5, 10 mM NaCl, 3 mM MgCl_2_, 0.1 mM EDTA, 0.5% NP-40, 0.2 mM Pefabloc SC and complete protease inhibitor cocktail (Roche)), and incubated on ice for 5 min. After centrifugation at 1800 rpm for 5 min at 4°C, supernatant (cytoplasm extract) was carefully removed. Nuclei pellets were washed once with MNase buffer (10 mM Tris, PH 7.5, 15 mM NaCl, 60 mM KCl, 0.15 mM spermine and 0.5 mM spermidine), and nuclei resuspended in the same buffer (at a density of 5 × 10^6 ^per 94 μL MNase buffer). MNase digestion was carried out in the presence of 2 mM CaCl_2 _and 50 U of MNase (micrococcal nuclease, NEB) for 10 min or the desired digestion time at 37°C. The reaction was stopped by the addition of 20 mL termination buffer. A QIAamp DNA blood kit (Qiagen) was used immediately after the MNase digestion for DNA isolation. The MNase digested samples were either subjected to gel electrophoresis (1% agarose gel) or quantitative real-time PCR. Real-time PCR were carried out by using primers of interest and Power SYBR® Green PCR Master Mix (ABI). The primer sequences were designed against both the promoter regions and 1 kb up-stream of the gene TSS, and are listed in Additional file 6. DNA accessibility was calculated based on a method described previously [56].

### Statistics

Data were expressed as mean ± SEM of at least three independent experiments. Statistical significance was computed by One Way ANOVA (SPSS 17.0). A *p *< 0.05 was considered statistically significant.

## Abbreviations

PMA/I: phorbol 12-myristate 13-acetate and calcium ionophore; FACS: fluorescent-activated cell sorting; MeDIP: immunoprecipitation of methylated DNA; IL2: interleukin 2; CSF2: granulocyte-macrophage colony-stimulating factor; DNMT: DNA methyltransferase; AICDA: activation-induced deaminase; APOBEC: apolipoprotein B-editing catalytic; Gadd45: growth arrest and DNA-damage-inducible 45; TET: Ten-eleven-translocation proteins.

## Competing interests

The authors declare that they have no competing financial interests. This work was funded by Bioplatforms Australia. This funding does not alter our adherence to all the BMC Molecular Biology policies on sharing data and materials.

## Authors' contributions

YL performed the experiments and prepared the manuscript; GC, LM and CS provided technical support; MFS and JF designed the project. JF and SO prepared the manuscript. All authors read and approved the final manuscript.

## Supplementary Material

Additional file 1 **Shift from naive CD4^+ ^T cells to memory and effector T subset cells during development**. *A. & B*. FACS analysis shows cell-surface phenotype of unstimulated CD4^+ ^T cells (NS) isolated from 2 week (*A*) and 16 week (*B*) old C57BL/6 mouse spleens gated at CD4^+^. Naive T cells are characterised by the presence of a CD44^-^CD62L^+ ^phenotype, while memory and effector T subset cells display a CD44^+^CD62L^+/- ^phenotype. *C. - D*. FACS analysis showing the cell-surface phenotype of PMA/I stimulated CD4^+ ^T cells (4 h) isolated from 2 week (*C*) and 16 week (*D*) old C57BL/6 mice. Activated cells become CD62L negative. Every experiment was repeated at least 3 times Click here for file

Additional file 2 **mRNA expression of inducible genes in CD4^+ ^T cells**. Total RNA was prepared from non-differentiated naive T cells, and differentiated Th1, Th2, Th17, nTreg and iTreg cells. After reverse transcription, quantitative PCR analysis was performed on cDNA with primers designed to detect inducible genes. Expression of *Ifng*, *Il4*, *Il17 *and *Foxp3 *in all the undifferentiated and differentiated T cells were normalized to the *Ubc *housekeeping gene. The data are shown as the mean and SEM of at least three independent experiments Click here for file

Additional file 3 **mRNA expression of inducible genes in CD4^+ ^T cells**. Total RNA was prepared from primary CD4^+ ^T cells, either unstimulated or stimulated with PMA/I for the indicated times. *A*. Expression of *Il2 *and *Csf2 *in primary CD4^+ ^T cells isolated from both age groups of mice was normalized to the *Ubc *housekeeping gene. *B & C*. Expression of *Il2 *and *Csf2 *in primary CD4^+ ^T cells isolated from 2 week (*B*) and 16 week (*C*) old C57BL/6 mice are presented as fold change relative to unstimulated cells in addition to initial *Ubc *normalization as mentioned in *A*. Normalized gene expression is shown on a log scale. All experiments were repeated at least 3 times Click here for file

Additional file 4 **FACS analysis of differentiated T helper cells**. FACS analysis shows cell-surface phenotype of differentiated Th1(*A*), Th2 (*B*), Th17 (*C*), nTreg (*D*) and iTreg (*E*) cells after intracellular staining with the corresponding antibodies, (*A*) anti-IFNg for Th1 cells, (*B*) anti-IL4 for Th2 cells, (*C*) anti-IL17 for Th17 cells or (*D*) anti-CD25 for nTreg and iTreg; all FACS analyses were gated at CD4^+^. All the differentiated cell subsets used in this study were purified by FACS sorting. All experiments were repeated at least 3 times Click here for file

Additional file 5 **Primers for gene expression**. Click here for file

Additional file 6 **Primers for MeDIP and CHART-PCR**. Click here for file
