# Supplementary figures and images for: Plasticity of DNA methylation in mouse T cell activation and differentiation
Source: BMC Mol Biol. 2012 May 29;13:16. doi: 10.1186/1471-2199-13-16 (PMC3386888; doi:10.1186/1471-2199-13-16)

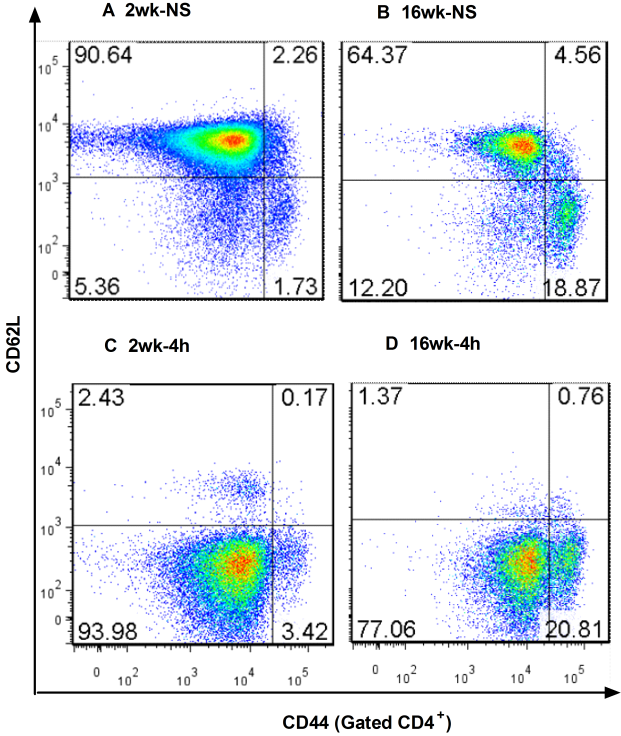

Supplement: Additional file 1 — Shift from naive CD4+ T cells to memory and effector T subset cells during development. A. & B. FACS analysis shows cell-surface phenotype of unstimulated CD4+ T cells (NS) isolated from 2 week (A) and 16 week (B) old C57BL/6 mouse spleens gated at CD4+. Naive T cells are characterised by the presence of a CD44-CD62L+ phenotype, while memory and effector T subset cells display a CD44+CD62L+/- phenotype. C. - D. FACS analysis showing the cell-surface phenotype of PMA/I stimulated CD4+ T cells (4 h) isolated from 2 week (C) and 16 week (D) old C57BL/6 mice. Activated cells become CD62L negative. Every experiment was repeated at least 3 times [file 1471-2199-13-16-S1.DOC]

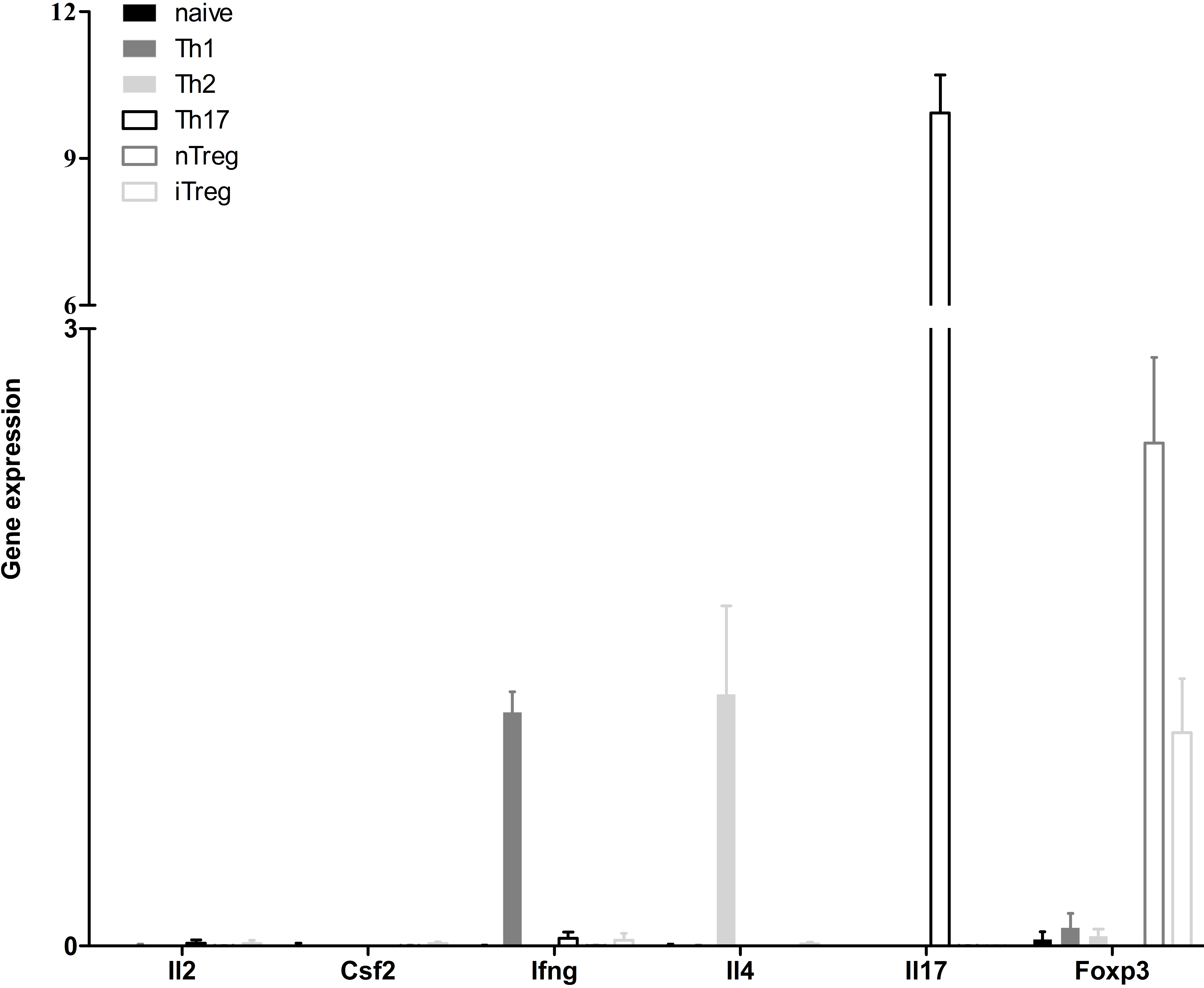

Supplement: Additional file 2 — mRNA expression of inducible genes in CD4+ T cells. Total RNA was prepared from non-differentiated naive T cells, and differentiated Th1, Th2, Th17, nTreg and iTreg cells. After reverse transcription, quantitative PCR analysis was performed on cDNA with primers designed to detect inducible genes. Expression of Ifng, Il4, Il17 and Foxp3 in all the undifferentiated and differentiated T cells were normalized to the Ubc housekeeping gene. The data are shown as the mean and SEM of at least three independent experiments [file 1471-2199-13-16-S2.DOC]

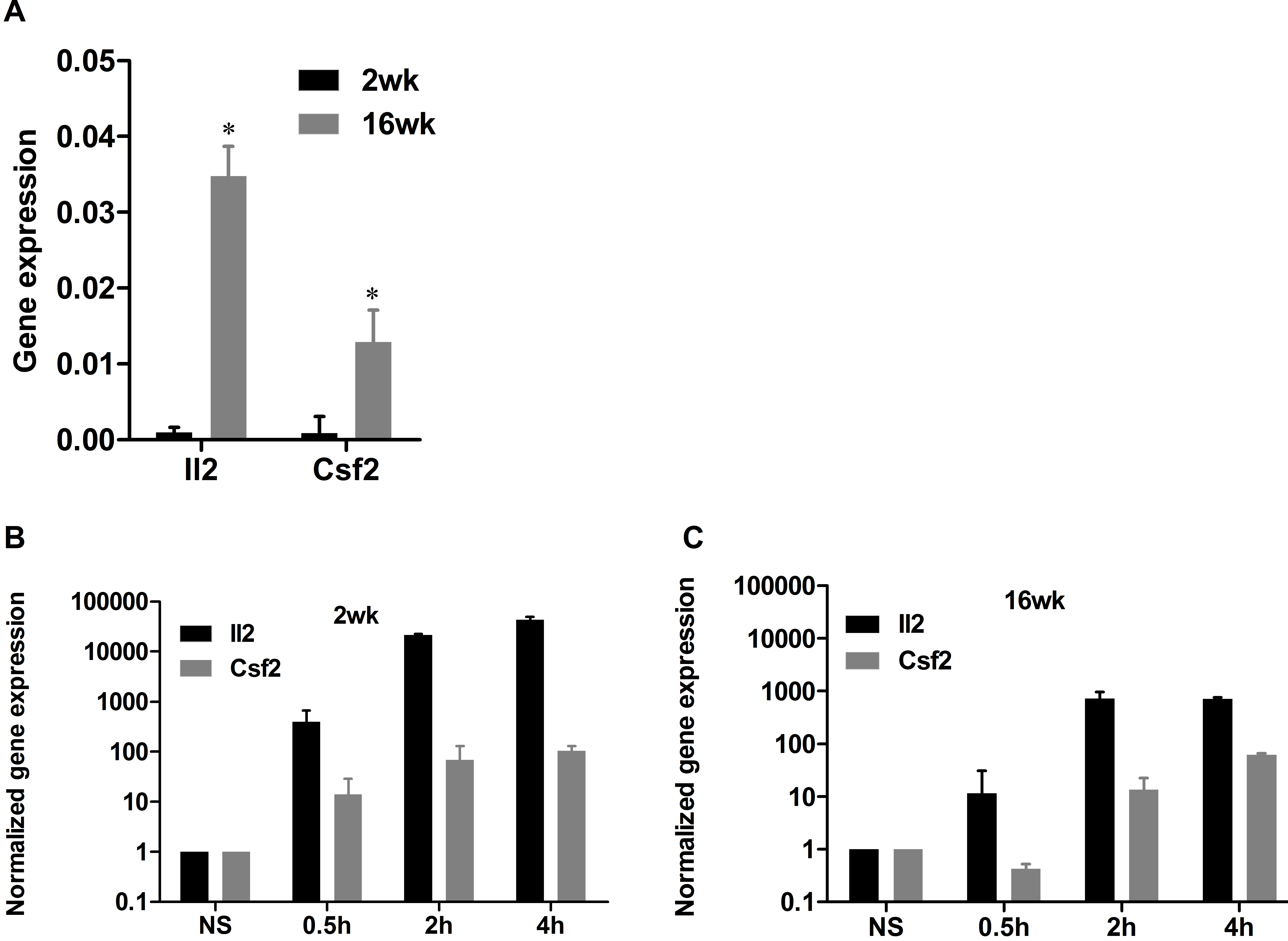

Supplement: Additional file 3 — mRNA expression of inducible genes in CD4+ T cells. Total RNA was prepared from primary CD4+ T cells, either unstimulated or stimulated with PMA/I for the indicated times. A. Expression of Il2 and Csf2 in primary CD4+ T cells isolated from both age groups of mice was normalized to the Ubc housekeeping gene. B & C. Expression of Il2 and Csf2 in primary CD4+ T cells isolated from 2 week (B) and 16 week (C) old C57BL/6 mice are presented as fold change relative to unstimulated cells in addition to initial Ubc normalization as mentioned in A. Normalized gene expression is shown on a log scale. All experiments were repeated at least 3 times [file 1471-2199-13-16-S3.DOC]

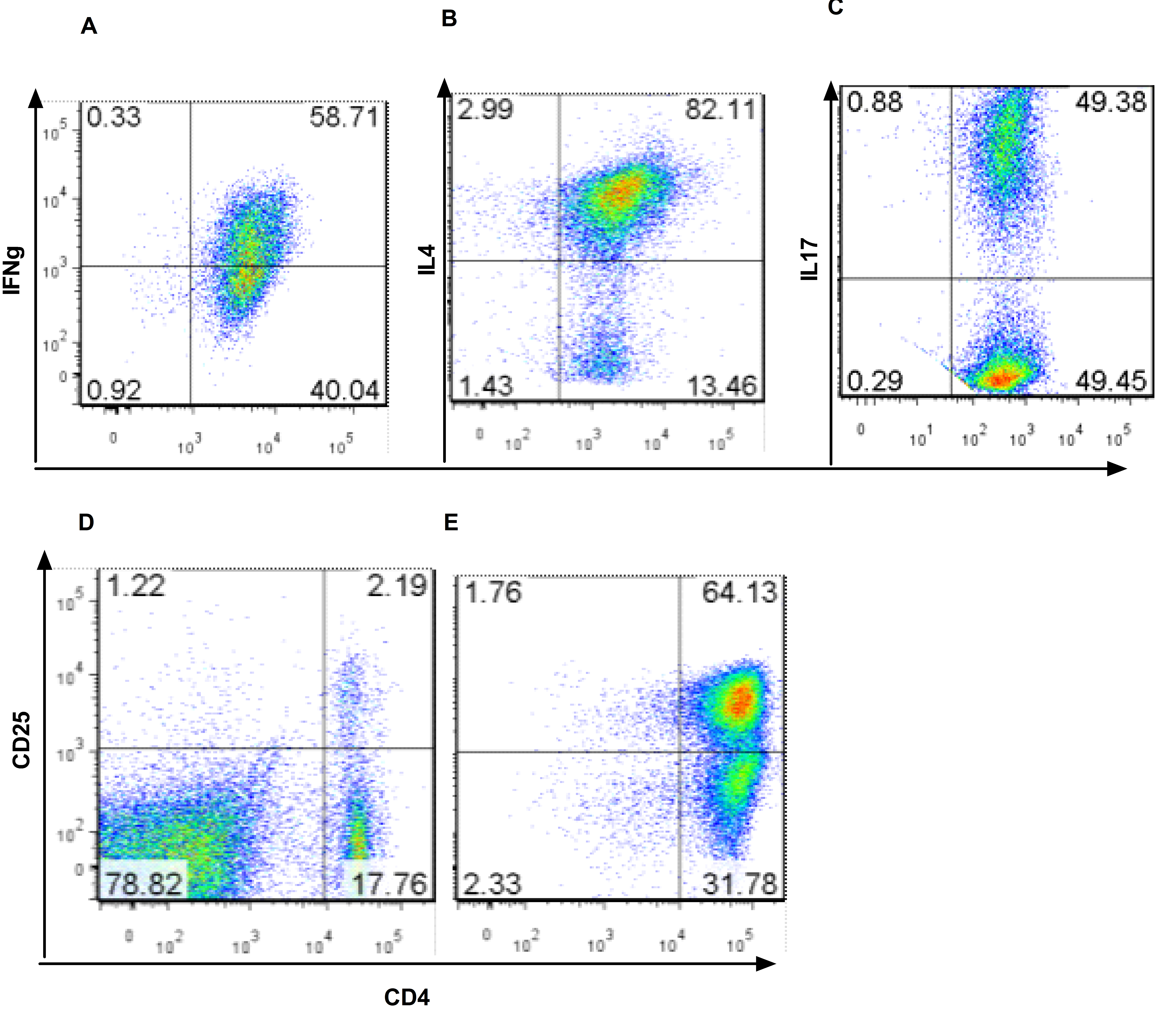

Supplement: Additional file 4 — FACS analysis of differentiated T helper cells. FACS analysis shows cell-surface phenotype of differentiated Th1(A), Th2 (B), Th17 (C), nTreg (D) and iTreg (E) cells after intracellular staining with the corresponding antibodies, (A) anti-IFNg for Th1 cells, (B) anti-IL4 for Th2 cells, (C) anti-IL17 for Th17 cells or (D) anti-CD25 for nTreg and iTreg; all FACS analyses were gated at CD4+. All the differentiated cell subsets used in this study were purified by FACS sorting. All experiments were repeated at least 3 times [file 1471-2199-13-16-S4.DOC]
