# Supplementary material for: Plasticity of DNA methylation in mouse T cell activation and differentiation
Source: BMC Mol Biol. 2012 May 29;13:16. doi: 10.1186/1471-2199-13-16 (PMC3386888; doi:10.1186/1471-2199-13-16)
Supplement: Additional file 5 — Primers for gene expression. [file 1471-2199-13-16-S5.DOC]

| **Gene** | **Forward/Reverse** | **Sequence** |
| --- | --- | --- |
| Ubc | For | 5’-AAGAGAATCCACAAGGAATTGAATG |
|  | Rev | 5’-CAACAGGACCTGCTGAACACTG |
| Il2 | For | 5’-CCTGAGCAGGATGGAGAATTACA |
|  | Rev | 5’-TCCAGAACATGCCGCAGAG |
| Csf2 | For | 5’-AAGGTCCTGAGGAGGATGTG |
|  | Rev | 5’-GAGGTTCAGGGCTTCTTTGA |
| Dnmt1 | For | 5’-AGGAGTGTGTGAGGGAG |
|  | Rev | 5’-GGTGTCACTGTCCGACTTGC |
| Dnmt3a | For | 5’-ACCCATGCCAAGACTCACCTTC |
|  | Rev | 5’-TCCACCTTCTGAGACTCTCCAG |
| Dnmt3b | For | 5’-TCAGACACGAAGGATGCTCC |
|  | Rev | 5’-ACAGGGTACTCCTGCACATG |
| Mbd4 | For | 5’-AAAGGACCTGGTGATAAGCAGT |
|  | Rev | 5’-GCTTCACAACTCTTTCCCATCCA |
| Tdg | For | 5’-AAGTTCCTAACATGGCAGTCAC |
|  | Rev | 5’-ATTTCTTCGACGTAGCAGGTTT |
| Apobec3 | For | 5’-CAACGTACAGGACCCAGAAAC |
|  | Rev | 5’-TCTTCACTTAGCGGGTCCATTCGC |
| Apobec4 | For | 5’-AACTGCCCTTACCACATTCG |
|  | Rev | 5’-AGCCTCGTTACAAGGGGAGT |
| Gadd45a | For | 5’-TGCTGCTACTGGAGAACGAC |
|  | Rev | 5’-CGACTTTCCCGGCAAAAACAAA |
| Gadd45b | For | 5’-AGACATTGGGCACAACCGAAG |
|  | Rev | 5’-CCATTGGTTATTGCCTCTGCT |
| Gadd45g | For | 5’-TTTCACGTTGATTCAGGCGTT |
|  | Rev | 5’-AAATGAGGATGCAATGCAGGT |
| Tet1 | For | 5’-AGTGAGGAGATCTACGCCGA |
|  | Rev | 5’-ACAAGGGACACACGAAAAGG |
| Tet2 | For | 5’-GGTTCTCAACGAGCAGGAAG |
|  | Rev | 5’-TGAGATGCGGTACTCTGCAC |
| Tet3 | For | 5’-TACAATGGGTGCACTGTGGT |
|  | Rev | 5’-CCACTACTGACCTTGGCGTT |
