# Supplementary material for: Plasticity of DNA methylation in mouse T cell activation and differentiation
Source: BMC Mol Biol. 2012 May 29;13:16. doi: 10.1186/1471-2199-13-16 (PMC3386888; doi:10.1186/1471-2199-13-16)
Supplement: Additional file 6 — Primers for MeDIP and CHART-PCR. [file 1471-2199-13-16-S6.DOC]

| **Gene** | **Forward/Reverse** | **Sequence** |
| --- | --- | --- |
| Il2-A | For | 5’-CACAGGTAGACTCTTTGAAAATATGTGTAA |
|  | Rev | 5’-CATGGGAGGCAATTTATACTGTTAATG |
| Il2-B | For | 5’-TCACCTAAATCCATTCAGTCAGTGTA |
|  | Rev | 5’-GTGGCAGAAAGCATTACCTTTG |
| Il2-C | For | 5’-CTTTTGTGTCTCCACCCCAAA |
|  | Rev | 5’-CACACTTAGGTGGCAGTTTTAATTCAT |
| Il2-F | For | 5’-CATGCAGAGTTTTTTGTTGTTTTCTAG |
|  | Rev | 5’-GCCTAAAGTCTCTCACAAAGAACAGA |
| Csf2-A | For | 5’-AAAAGGAGAGGCTAGCCAGA |
|  | Rev | 5’-TAAGCCCTTCCAAGAACTGG |
| Csf2-B | For | 5’-TGGAATGAGCCACCAGAGTA |
|  | Rev | 5’-GGCTCTTGCTTCCATAGCAC |
| Il4-A | For | 5’-AGGTGTGCTCAAGGCAGACT |
|  | Rev | 5’-GCCAATCAGCACCTCTCTTC |
| Ifng-A | For | 5’-CCACAAGAATGGCACAGGT |
|  | Rev | 5’-TACCTGATCGAAGGCTCCTC |
| Il17-A | For | 5’-CGAGACAGATGTTGCCCG |
|  | Rev | 5’-CGTGCAATAACGCAACCTG |
| Il17-B | For | 5’-ACCTCATGTCTCTCGCTACTCC |
|  | Rev | 5’-TTTAACACCAACGGAACACG |
| Foxp3-A | For | 5’-AGCGAGGTCTGCGGCTTCC |
|  | Rev | 5’-CCACTTTCGCCAGCAGAGCT |
| Foxp3-B | For | 5’-CGATCCTCCAACGTCTCAC |
|  | Rev | 5’-CGGAAATTTTGAAATTTTGGG |
